# Effect of Duplicate Genes on Mouse Genetic Robustness: An Update

**DOI:** 10.1155/2014/758672

**Published:** 2014-07-10

**Authors:** Zhixi Su, Junqiang Wang, Xun Gu

**Affiliations:** ^1^State Key Laboratory of Genetic Engineering and MOE Key Laboratory of Contemporary Anthropology, School of Life Sciences, Fudan University, Shanghai 200433, China; ^2^Department of Genetics, Development & Cell Biology, Iowa State University, Ames, IA 50010, USA

## Abstract

In contrast to *S. cerevisiae* and *C. elegans*, analyses based on the current knockout (KO) mouse phenotypes led to the conclusion that duplicate genes had almost no role in mouse genetic robustness. It has been suggested that the bias of mouse KO database toward ancient duplicates may possibly cause this knockout duplicate puzzle, that is, a very similar proportion of essential genes (*P*_*E*_) between duplicate genes and singletons. In this paper, we conducted an extensive and careful analysis for the mouse KO phenotype data and corroborated a strong effect of duplicate genes on mouse genetics robustness. Moreover, the effect of duplicate genes on mouse genetic robustness is duplication-age dependent, which holds after ruling out the potential confounding effect from coding-sequence conservation, protein-protein connectivity, functional bias, or the bias of duplicates generated by whole genome duplication (WGD). Our findings suggest that two factors, the sampling bias toward ancient duplicates and very ancient duplicates with a proportion of essential genes higher than that of singletons, have caused the mouse knockout duplicate puzzle; meanwhile, the effect of genetic buffering may be correlated with sequence conservation as well as protein-protein interactivity.

## 1. Introduction

Functional compensation of duplicate (paralogous) genes has been thought to play an important role in genetic robustness [1–7]. Indeed, existence of a close paralog in the same genome could result in null mutations of the gene with little effect on the organismal fitness (nonessential gene), as observed in both yeast and nematode [1–4]. However, the role and magnitude of the duplicate genes contributing to genetic robustness in mammals remain controversial [8–13]. Two studies on mouse knockout phenotypes [9, 10] observed that the proportion of essential genes (*P*_*E*_) is similar between duplicate genes and singletons in mouse, sharply contrasted to those well-known findings that removing a duplicate gene usually generates less deleterious phenotypes than removing a single-copy gene [1–4]. On the other hand, Hsiao and Vitkup [8] suggested an important role in robustness against deleterious mutations of duplicate genes in human [8]. We call this controversy the knockout duplicate puzzle in mammals. Since knockout mice have been widely used as animal models for human diseases, resolving this issue may have a significant impact on biomedical sciences.

In summary, there are three alternative hypotheses proposed.

*(i) The Duplicability Hypothesis*. By combining the protein-protein interaction data into the analysis, Liang and Li [9] found that mouse duplicate genes tend to have much higher protein connectivity than those for singletons. Since high connectivity means high functional centrality in the gene network, they proposed that mouse duplicates probably are more important than singletons and that this factor could compromise the contribution of duplicate compensation. In other words, functionally important genes may have more chance to be duplicated. It remains unexplained why more important mouse genes tend to be duplicated, while yeast genes may have the opposite trend [14].

*(ii) The No-Role Hypothesis*. In contrast, Liao and Zhang [10] argued that the compensational role of duplicates in mouse genetic robustness is negligible. After examining a number of genomic factors, they discussed several possibilities that may result in similar proportion of essential genes between singletons and duplicates. It implies that most recently duplicated mouse genes, for example, 26 rodent-specific prolactin-like proteins [15], may have lost functional compensations to each other. This prediction seems to be counterintuitive and does not receive much experimental evidence for supporting.

*(iii) Age-Distribution Hypothesis*. Su and Gu [11] have noticed the effect of sampling bias: recently duplicated genes, for example, after the mammalian radiation, are severely underrepresented in the current mouse KO database. Because most of the mouse gene knockouts were generated by individual laboratories for finding knockout phenotypes, recently duplicated genes may have been purposely avoided to minimize the experimental cost due to negative-phenotype results. In other words, the age distribution of duplicates in the data sample is upwardly biased, resulting in underestimation of the overall duplicate effect on the genetic robustness.

*(iv) The Functional Importance Hypothesis*. Makino et al. (2009) reported that there is a strong sampling bias towards the duplicated genes generated by whole genome duplication (WGD) in current mouse KO phenotype dataset [12].

Since most of the mouse WGD duplicates are ancient duplicate genes, their conclusion that the mouse knockout duplicate puzzle may be caused by sampling bias of WGD duplicate genes is consistent with age-distribution hypothesis. Previous studies [16–18] have shown that mammalian duplicate genes can be characterized as two waves (Wave-I for young duplicates and Wave-II for those duplicated around the origin of vertebrates) and the ancient component (prior to the split of vertebrates and* Drosophila*). We [11] observed that the mouse (Wave-I) young duplicates were indeed severely underrepresented, and, for duplicates in the knockout experiments, their characteristic age (duplication time) could be as ancient as that of Wave-II (early vertebrates) or even more ancient. Obviously, very ancient duplicates certainly have little effect on the genetic robustness. However, due to the space limit, in the short communication we only had a brief discussion about the other two hypotheses. In this paper, we conducted an extensive and careful analysis for the updated mouse gene deletion phenotype data to evaluate the relative merit between the duplicability hypothesis, the no-role hypothesis, and the age-distribution of duplicates hypothesis.

In this paper, we use an updated mouse KO dataset to carry out an extensive analysis. To facilitate the study, we proposed an empirical evolutionary model of gene essentiality—the A&B model (Age of duplication and genetic Buffering)—to explain knockout duplicate puzzle. Our results suggest that duplication age and genetic buffering determine the essentiality of mouse duplicates.

## 2. Results

### 2.1. Similar *P*_*E*_ between Singletons and Duplicates Caused by Strong Bias in Mouse KO Genes toward Ancient Duplicates

Of the 4123 mouse genes with available phenotypic data, 1921 were identified as essential genes. Meanwhile, we identified 2479 duplicate genes and 464 singleton genes and calculated proportions of essential genes (*P*_*E*_), respectively. Consistent with previous studies [9–12], the updated mouse KO dataset shows no statistical difference of *P*_*E*_ between singletons and duplicates (44.8% versus 46.3%; *P* = 0.56). That is, proportions of essential genes in mouse singletons and duplicates are similar, in contrast to the well-known observations in other model organisms [1, 3]. Based on a more broad definition of gene essentiality (Materials and Methods), that is, genes with premature death or induced morbidity phenotype were considered as essential genes, we found the same pattern (data not shown).

**Figure 1:**
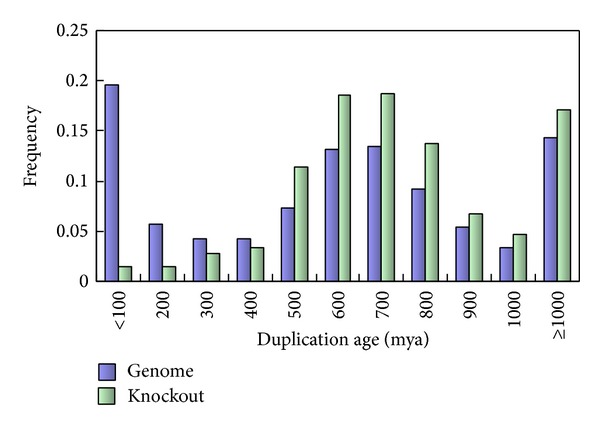
Duplication age distribution of mouse genome set (blue bars) and knockout gene set (green). The* x*-axis indicates the duplication age (*t*) between a duplicated gene and its closest paralog. The* y*-axis indicates the frequency of the duplicates in each duplication age category.

Though it is highly suspected that recently duplicated genes may have been underrepresented in the mouse KO database, detection of such bias at the genome level has been shown to be nontrivial [9–11], and Su and Gu [11] proposed a practically feasible solution: estimate the age of duplication event from the assumption of molecular clock. Since time estimation is well known to be error prone and based on a number of assumptions [19, 20], we have to develop a robust analytical pipeline to minimize the potential errors (see Materials and Methods). As shown in Figure 1, the histogram of mouse duplication events, short for the genome set, has recaptured the unique evolutionary feature of vertebrate gene families [16]. That is, it shows a pattern characterized by two waves (I, II) and an ancient (III) component [21, 22].

In the same manner, we estimated the duplication times between 2260 mouse knockout genes and their closest paralogs and found that the age distribution of duplicate pairs differs significantly between the genome set and the knockout set (*P* < 10^−16^,*χ* ^2^-test). The histograms in Figure 1 clearly show that mouse KO experiments have been designed to avoid recently duplicated genes, for example, only 1.4% for those duplicated within 100 mya (around or after the mammalian radiation) in the KO set, compared to 19.6% in the mouse genome set. Consequently, the ages of duplicate genes in the mouse knockout dataset are typically around 500 to 700 mya (in early vertebrates), with a long-tail toward even more ancient ones (>1000 mya). In other words, the sampling bias toward ancient duplicates in the currently available mouse KO target genes has been nontrivial. These ancient duplicates may have undergone substantial functional divergence so that they have lost the capacity of functional compensation. In contrast, recent gene duplications, those duplicated around the mammalian radiation or in the rodent lineage, are expected to have significant contributions to the gene robustness in the current mouse genome. While these young duplicates were considerably underrepresented in the mouse knockout dataset, the observed proportion of essential duplicate genes is upwardly biased close to the value of singletons. 

### 2.2. The Duplication-Age and Buffering Model (Age-Buffering Model) of Gene Essentiality

Since initially duplicated genes were completely compensated, the loss process of duplicate compensation is apparently time dependent, during which the outcome can be influenced by many gene-specific factors. To have a complete understanding of gene essentiality in duplicates and singletons, an evolutionary model is needed. We formulate a simple* A&B* model as follows, short for* A*ge of duplication and genetic* B*uffering. Without genetic buffering, we assume that the probability of a duplicate remains nonessential, that is, functionally compensated by another duplicate copy in the same genome, and decayed exponentially with the time* t* (the age of gene duplication), that is,  *e* ^−*λt*^, where *λ* is the loss rate of duplicate compensation by mutations. Next, let *g* be the probability that a gene is genetically buffered. Together, the* A&B* model demonstrates that a gene to be essential depends on two mechanisms: the effect of genetic buffering (*g*) and the age-dependent effect of duplication compensation (*e* ^−*λt*^). Obviously, the probability of a duplicate gene being essential is the probability for both mechanisms failure, that is,

(1) $$\begin{array}{c} P_{E}=\left(1-g\right)\left(1-e^{-\lambda t}\right). \end{array}$$

Under this model, the negligible role hypothesis [10] actually claimed a very high loss rate (*λ*) of functional compensation such that *P*_*E*_ ≈ *P*_E_* in the current mouse genome. On the other hand, the duplicability model [9] assumes that the effect of genetic buffering (*g*) of duplicates is lower than that of singletons denoted by g*, that is, *g* < g*, such that (1 − *g*)(1 − *e* ^−*λt*^) ≈ 1 − g* holds. In fact, (1) suggests that three parameters,* t* (duplication age), *g* (genetic buffering), and *λ* (loss rate of functional compensation), together determine the gene essentiality of mouse duplicates. Particularly, we have two claims: (i) the proportion of essential genes in mouse duplicates (*P*_*E*_) is age dependent on gene duplications; (ii) gene essentiality correlates to sequence conservation or protein connectivity in either duplicates or singletons largely because these two factors affect the efficiency of genetic buffering (*g*), rather than the functional compensation between duplicates. Our preliminary analysis [11] has shown the first claim. In the following we provide a detailed analysis to address some technical issues and doubts.

Our models suggested that, for sufficient time, *P*_*E*_ approaches to a level that is roughly equal to *P*_*E*_ of singleton. However, it does not mean that all these ancient duplicates are subject to the genetic buffering. A likely situation is that genetic buffering and duplication coevolve. In other words, the reason why some duplicates can remain dispensable for a long time is because they were integrated into existing or novel genetic buffering mechanisms. 

Chen et al. (2010) found that in* Drosophila* new genes could become essential rapidly after the gene duplications [23]. This mechanism is also likely to exist in mammals. To take this factor into account, we modify (1) as follows:

(2) $$\begin{array}{c} P_{E}=\left(1-g\right)\left[1-\left(1-\rho \right)e^{-2\lambda t}\right], \end{array}$$

where the parameter *ρ* > 0 indicates the process of rapid essentiality in the early stage after gene duplication. Because the number of mouse KO genes is small for very young duplicates, a further investigation requires when the data are available.

### 2.3. Proportions of Essential Genes (*P*_*E*_) in Mouse Duplicates Are Age Dependent

**Figure 2:**
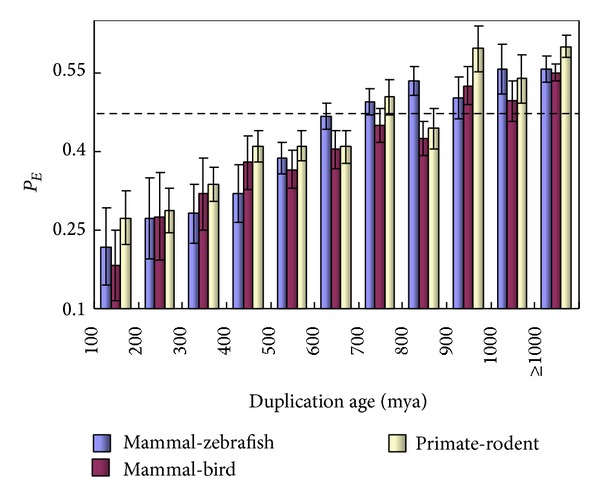
Relationship between *P*_*E*_ in duplicate genes and the duplication age. Error bars show one standard error. The dashed line indicates the *P*_*E*_ level of single-copy genes.

A simple solution to correct this knockout sampling bias is to calculate *P*_*E*_ under a given age bin. We implemented several approaches to minimize the noise effect in time estimation. First, we used three time calibration points to date mouse duplication events: the mammal-zebrafish split (430 mya), the mammal-bird split (310 mya), and the primate-rodent split (80 mya), respectively, and calculated *P*_*E*_ for every age bin of 100 million years. As shown in Figure 2, in all cases we observed that *P*_*E*_ increases from a low value in young duplicates with the increasing of duplication ages; this *P*_*E*_-age (*t*) correlation is statistically significant (*P* < 10^−4^, *χ* ^2^-test). To be concise, in the following of this paper, we mainly present the results based on the mammal-zebrafish split time calibration. Noticeably, we found that *P*_*E*_ in ancient duplicates, say, >700 mya, is unexpectedly higher than that of singletons;* P*_E_ = 0.542 ± 0.016,* P* < 0.001. Hence, there are two reasons for why the overall *P*_*E*_ in duplicates has no difference from that of singletons: the sampling bias toward ancient duplicates and very ancient duplicates with a higher *P*_*E*_ than that of singletons. In addition, we conducted simulations to examine the effect of violation of molecular clock (constant evolutionary rate) on the estimation of *P*_*E*_. Our results showed that the age dependency of *P*_*E*_ can be weakened or even vanished by the violation of molecular clock. In other words, our conclusion of *P*_*E*_-age correlation seems to be conserved (not shown). Finally, we inferred the phylogenetic locations of mouse KO duplication events in three intervals: after the mammal-zebrafish split, after the mammal-bird split, and after the primate-rodent split. In each interval we calculated *P*_*E*_, which is compatible to the proportion of essential genes, with respect to the three major speciation events in vertebrates: *P*_*E*_ is ~23% for those duplicated after the mammalian radiation, ~31% for those duplicated after the bird-mammal split, and close to ~39% for those duplicated after the teleost-tetrapod split. Although a decreasing *P*_*E*_ in younger duplicates is biologically intuitive, it is subject to the statistical uncertainty due to small sample size. Nevertheless, under a more broad age category, such as before the split of land animals and fishes versus the more ancient duplicates, the difference is statistically significant (*P* < 0.01).

In a separate study, we developed a simple bias-correcting procedure to obtain a bias-corrected *P*_*E*_ and test whether it is significantly lower than in singletons. We predicted that *P*_*E*_ *=* 41.7% for all duplicate genes, which are impressive compared to *P*_*E*_ *= *46.3% observed in sample duplicates and *P*_*E*_ = 47% in sample singletons [11]. However, in this study, when we used a more stringent criterion to define single-copy genes, we found that there is no statistical significant difference between the predicted *P*_*E*_ and *P*_*E*_ of single-copy genes (41.7% versus 44.8%, *P* = 0.21). We want to emphasize that, even after taking this bias into consideration, the difference between *P*_*E*_ for singletons and *P*_*E*_ for duplicates at the genome level is still small. This may be because the contribution of functional compensation by young duplicates cancels the contribution of higher intrinsic importance of ancient duplicate, which is consistent with the duplicability hypothesis [9].

### 2.4. Age Dependence of *P*_*E*_ in Mouse Duplicates and Sequence Conservation

Though a simple interpretation for the *P*_*E*_-*t* correlation is that the capability of duplicate compensation decays with the evolutionary time since the duplication [11], some other alternatives cannot be ruled out, which were based on the correlation of gene essentiality with, for instance, sequence conservation or protein connectivity [9, 10, 24]. We have addressed these issues carefully.

**Figure 3:**
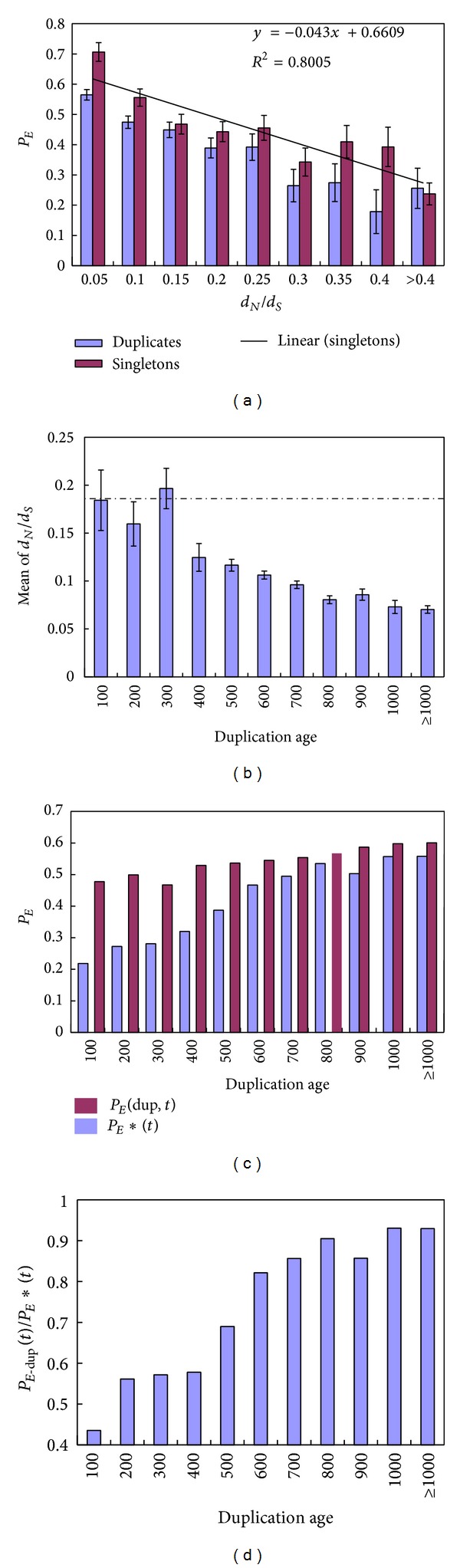
The effect of sequence conservation on the relationship between *P*_*E*_ and duplication age. (a) Relationship between *P*_*E*_ in duplicate genes (blue) or singletons (purple) and the evolutionary conservation of the gene, measured by the ratio of the nonsynonymous (*d*_*N*_) to synonymous (*d*_*S*_) nucleotide distances between the target gene and its human ortholog. Linear regression line and regression equation between *d*_*N*_/*d*_*S*_ ratio and *P*_*E*_ in knockout single-copy genes are presented on the panel. (b) Mean *d*_*N*_/*d*_*S*_ ratio for each age bin of duplicates. Dashed line denotes the mean *d*_*N*_/*d*_*S*_ ratio of singleton mouse knockout genes. (c) *P*_*E*_ in each age bin of duplicates—*P*_*E*_ (dup,* t*)—and that of singletons with the same *d*_*N*_/*d*_*S*_ ratio—*P*_E_*(*t*). *P*_E_*(*t*) is calculated based on the mean *d*_*N*_/*d*_*S*_ ratio for duplicates in each age bin (panel b) and the linear regression equation (panel a). (d) Ratio of *P*_*E*_ (dup,* t*) and *P*_E_*(*t*) in each age bin of duplicates. Error bars show one standard error.

To measure the sequence conservation, we used the conventional ratio of the number of nonsynonymous substitutions per site (*d*_*N*_) to the number of synonymous substitutions per site (*d*_*S*_), which was estimated from the mouse gene and its human ortholog (see Materials and Methods). A low *d*_*N*_/*d*_*S*_ ratio indicates high sequence conservation of the gene. Consistent with previous studies [10, 25], we showed that essential mouse genes tend to be more conserved: *P*_*E*_ decreases with the increase of *d*_*N*_/*d*_*S*_ for both duplicates (Spearman rank*ρ* = −0.23, *P* < 10^−15^) and singletons (*ρ* = −0.18, *P* < 10^−15^; see Figure 3(a) for binned results). After calculating the mean *d*_*N*_/*d*_*S*_ ratio for each age bin of mouse duplicates, we unexpectedly found that sequence conservation is actually positively correlated with the duplication age (*t*) (Figure 3(b), *P* < 10^−10^). This unexpected inverse age-*d*_*N*_/*d*_*S*_ relationship raises the possibility that the observed *P*_*E*_-*t* (age) correlation could be confounded by the*P*_*E*_-*d*_*N*_/*d*_*S*_ correlation conjugated with the age-*d*_*N*_/*d*_*S*_ correlation.

We first claim that the *P*_*E*_-*d*_*N*_/*d*_*S*_ correlation is the consequence of the inverse relationship between the genetic buffering (*g*) and the sequence conservation (*d*_*N*_/*d*_*S*_). Hence, the inverse age-*d*_*N*_/*d*_*S*_ relationship in mouse duplicates suggests less effect of genetic buffering in ancient duplicates than that in recent duplicates, implying that the genetic buffering of duplicates *g* could be age dependent. One possible evolutionary mechanism for the age-*g* inverse relationship could be the neofunctionalization in the late stage after the gene duplication so the preexisting (ancestral) genetic buffering systems did not work for the newly acquired functions.

Suppose that the effects of genetic buffering (*g*) are similar between singletons and duplicates, as long as they have a similar *d*_*N*_/*d*_*S*_ ratio; we designed a simple procedure as follows to take the effect of sequence conservation into account. That is, for each age bin (*t*) of duplicates, the buffering effect (1 − *g*) was estimated from the *P*_*E*_ of the singleton mouse KO genes, corrected by the linear regression with the *d*_*N*_/*d*_*S*_ ratio, and denoted by  *P*_E_*(*t*) (Figure 3(a)). To be clear, we used *P*_*E*-dup_(*t*) for the age-bin (*t*) of mouse duplicates.  Figure 3(c) plotted both *P*_*E*-dup_(*t*) and *P*_E_*(*t*) against age bins of duplicates. As expected, *P*_E_*(*t*) increases with the duplication age* t,* but much slower than*P*_*E*-dup_(*t*), indicating that the *P*_*E*_-*d*_*N*_/*d*_*S*_ correlation can only explain a small portion of the *P*_*E*_-age correlation in duplicates. According to (1), the relative essentiality in duplicates, *P*_*E*-dup_(*t*)/*P*_E_*(*t*), is given by

(3) $$\begin{array}{c} \frac{P_{E\text{-dup}}\left(t\right)}{P_{E}^{*}\left(t\right)}=1-e^{-\lambda t}, \end{array}$$

which measures the pure duplication effect on gene essentiality and does not depend on the sequence conservation. Indeed, we found a significantly positive correlation between the ratio *P*_*E*-dup_(*t*)/*P*_E_*(*t*) and the duplication age (*P *<* *0.001; Figure 3(d)). We repeated our analysis using *d*_*N*_/*d*_*S*_ ratio of mouse-rat orthologous gene pairs and obtained a virtually same result (Figure S1; see Figure S1 in Supplementary Material available online at http://dx.doi.org/10.1155/2014/758672). We therefore conclude that the proportion of essential genes (*P*_*E*_) of mouse duplicates is age dependent, even after correcting the potential confounding effect from the essentiality-conservation dependence. 

### 2.5. Age Dependence of *P*_*E*_ in Mouse Duplicates and Protein Connectivity

**Figure 4:**
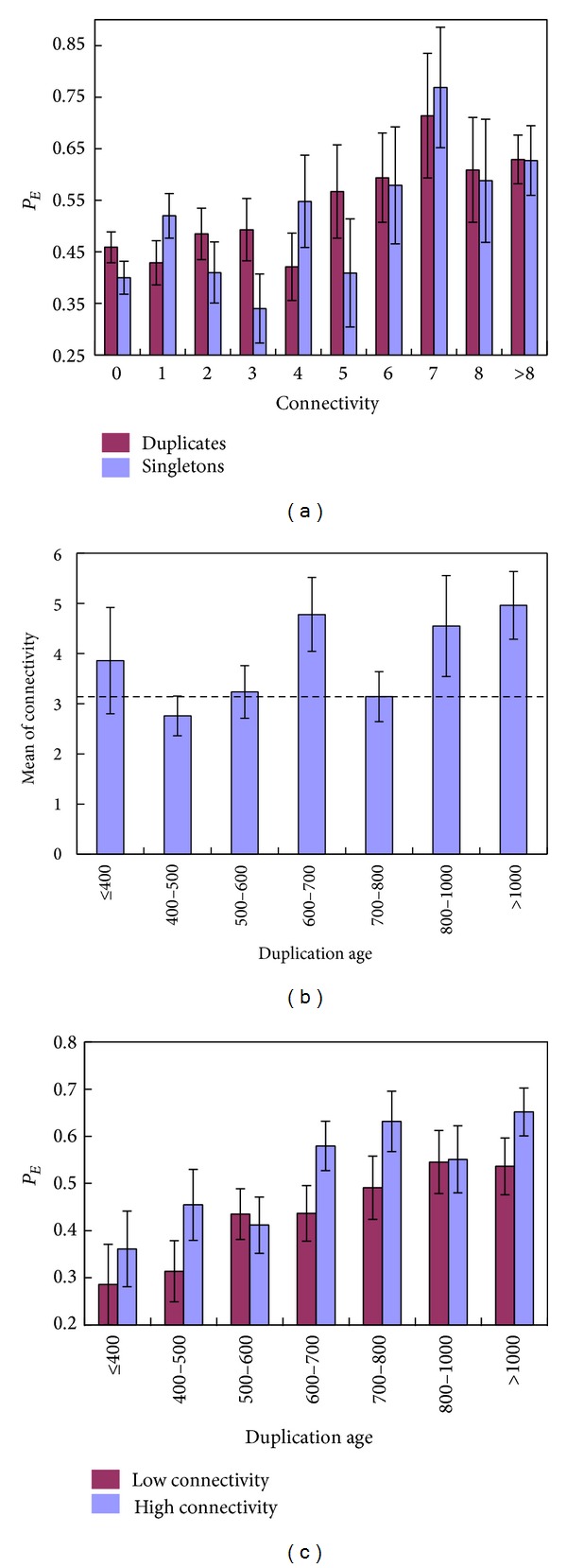
The effect of protein connectivity on the relationship between *P*_*E*_ and duplication age. (a) Relationship between *P*_*E*_ in duplicate genes (blue) or singletons (purple) and the protein connectivity of the gene. (b) Mean interaction number for each age bin of duplicates. Dashed line denotes the mean interaction number of singleton mouse knockout genes. (c) Relationship between *P*_*E*_ in duplicate genes and the duplication age for high connectivity genes and low connectivity genes. Error bars show one standard error.

The proportion of essential genes is positively correlated with protein connectivity in mouse [9]. In our updated mouse KO dataset, we compiled 211 singleton mouse KO targeted genes with available protein connectivity data, as well as 845 mouse KO duplicates [26]. Consistent with [9], we confirmed a weak but significant positive correlation between protein connectivity and *P*_*E*_ in both duplicates (Spearman rank*ρ* = 0.11, *P* = 0.001) and singletons (*ρ* = 0.11, *P* = 0.003; see Figure 4(a) for binned results). Similar to the effect of sequence conservation, the A&B model interprets this finding as genes with high connectivity may have low genetic buffering. Due to the small sample size, we further group the 845 genes into seven age groups. We then calculated the mean of protein interaction number for duplicated genes in each age bin and found no correlation of the mean protein connectivity with the duplication age (*t*) (Spearman rank *ρ* = 0.04, *P* = 0.19, Figure 4(b)).

We thus hypothesize that *P*_*E*_-connectivity and *P*_*E*_-age correlations reflect two independent underlying mechanisms. To further test this hypothesis, we divided duplicate genes with interaction data into two groups, those with high connectivity (larger than the median interaction, i.e., >2 interactions) and those with low connectivity (otherwise). The proportion of essential genes in the high-connectivity group is apparently higher than that in the low-connectivity group (*P* < 0.001). But, as shown in Figure 4(c), the inverse relationship between *P*_*E*_ and the age of duplicates holds in both gene groups. We thus conclude that age dependence of the proportion of essential genes (*P*_*E*_) in duplicates is unlikely to be confounded by the effect of protein connectivity.

### 2.6. Age Dependence of *P*_*E*_ Is Irrespective of Sampling Bias toward Essential Genes, Developmental Genes, or WGD Duplicates

It was proposed that individual researchers might tend to report a gene with a discernible phenotype in the KO experiments [10, 12]. Therefore, reports of gene knockouts with stronger phenotype (essential genes) are likely to be dramatically overrepresented in the KO dataset. A previous study found that the developmental genes and duplicated genes generated by WGD tend to be more essential than the nondevelopmental genes and small-scale duplication (SSD) duplicated genes, respectively. Besides, the current mouse KO dataset is biased toward developmental genes and WGD duplicates. Therefore, it is suspected that the ancient duplicates bias of KO duplicates and *P*_*E*_-*t* correlation might be only a byproduct of the above factors. Here, we tested whether the bias of ancient duplicates of KO dataset is a side effect of the biased sampling of WGD genes or developmental genes and whether age dependency of *P*_*E*_ still holds after controlling the influences of the above factors.

**Figure 5:**
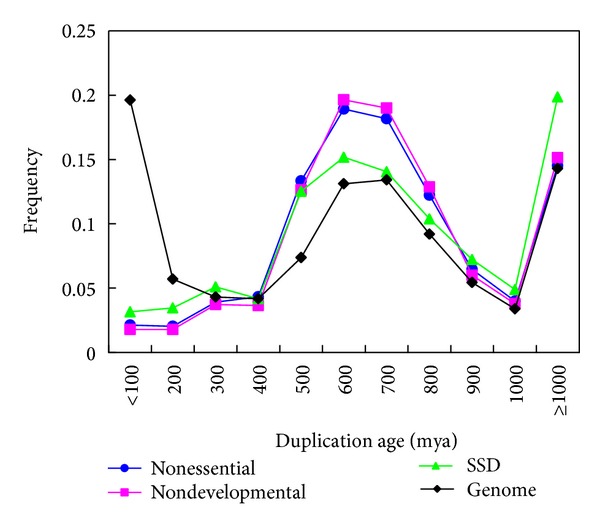
Duplication age distribution of mouse genome set (black), nonessential duplicates, nondevelopmental duplicates, and SSD duplicates.

If the sampling bias towards the ancient duplicates is just caused by the preferential report of the essential genes by individual mouse KO experiments, no age distribution difference would be expected between KO nonessential duplicates and the whole genome set. We then compared the age distribution of nonessential KO duplicates with the whole genome set. As shown in Figure 5, even after removing all essential genes, the KO duplicates still show strong age bias toward ancient duplicated genes. Therefore, we conclude that the age bias of KO genes is not an artifact of sampling bias of essential genes.

To test the influence of the sampling bias of developmental genes, we subdivided all the mouse genes with at least one GO item as developmental genes and nondevelopmental genes, based on the approaches of [12]. In the KO dataset, we found that the *P*_*E*_ of developmental genes is significantly higher than the *P*_*E*_ of nondevelopmental genes (66.1% versus 34.8%, *P* < 2.2*e* − 16, *χ* ^2^ test). For all of the duplicate genes with at least one GO item in KO dataset, we found 36.8% of them belonging to developmental genes, which is significantly larger than the proportion of developmental genes in whole genome set (13.4%, *P* < 2.2*e* − 16). The similar bias also has been found in single-copy genes (28.9% versus 8.3%, *P* < 2.2*e* − 16). These findings indicate that developmental genes were enriched in the mouse KO dataset irrespective of single-copy genes or duplicated genes, which is consistent with previous study [12]. If the sampling bias of KO duplicates toward the ancient duplicated genes is only caused by the bias of developmental genes, it is expected that the age distribution of KO nondevelopmental duplicates will be similar to that of whole genome set. However, for the nondevelopmental duplicates, we found that the age distribution of duplicates differs significantly between the genome set and KO set. That is, recently duplicated nondevelopmental genes have been underrepresented in the mouse nondevelopmental KO dataset (Figure 5). Since developmental genes are more essential than other genes, it is reasonable to suspect that the positive *P*_*E*_-*t* correlation might be simply because of the trend that ancient duplicates have more developmental genes. To address this issue, we calculated the *P*_*E*_-*t* correlation for developmental and nondevelopmental genes, respectively. We found that the *P*_*E*_-*t* correlation is statistically significant, in both developmental genes (*ρ* = 0.1, *P* = 0.002, Spearman rank test) and nondevelopmental genes (*ρ* = 0.2, *P* < 1*e* − 5).

The sampling bias of WGD duplicates also may confound our analysis. More and more evidences indicated that there may have been two rounds of WGD that occurred during the early stage of vertebrate evolution (500–700 mya), and duplicate developmental genes created by WGD were preferentially retained in vertebrate genome [12, 27]. We tested if we rule out the influence of WGD duplicates the A&B model still holds. Following the methods of [12], we obtained a list of human duplicated genes created by WGD inferred by [28]. We then inferred the mouse duplicated genes generated by whole genome duplication through one-to-one orthology relationships with the human genes. We identified 1237 mouse WGD duplicated genes and 1242 SSD duplicated genes with phenotype data. We found that the *P*_*E*_ of WGD duplicates is 51.1%, which is larger than the *P*_*E*_ of singletons (44.7%, *P* = 0.02). We then estimated the duplication age between all SSD duplicated KO genes and their closest paralogs and found that the age distribution of SSD duplicates still differs significantly between the genome set and SSD KO set (*P* < 1*e* − 16, *χ* ^2^ test). Figure 5 clearly shows that, even after ruling out the WGD genes, the KO duplicates dataset is still biased toward ancient duplicates. We further calculated the *P*_*E*_ for each bin of age (100 mya) and observed that *P*_*E*_-*t* correlation holds for SSD KO genes (*ρ* = 0.21, *P* < 1*e* − 11).

### 2.7. What Determines Duplicate Compensation: Evolutionary Time (Age) or Sequence Conservation? 

**Figure 6:**
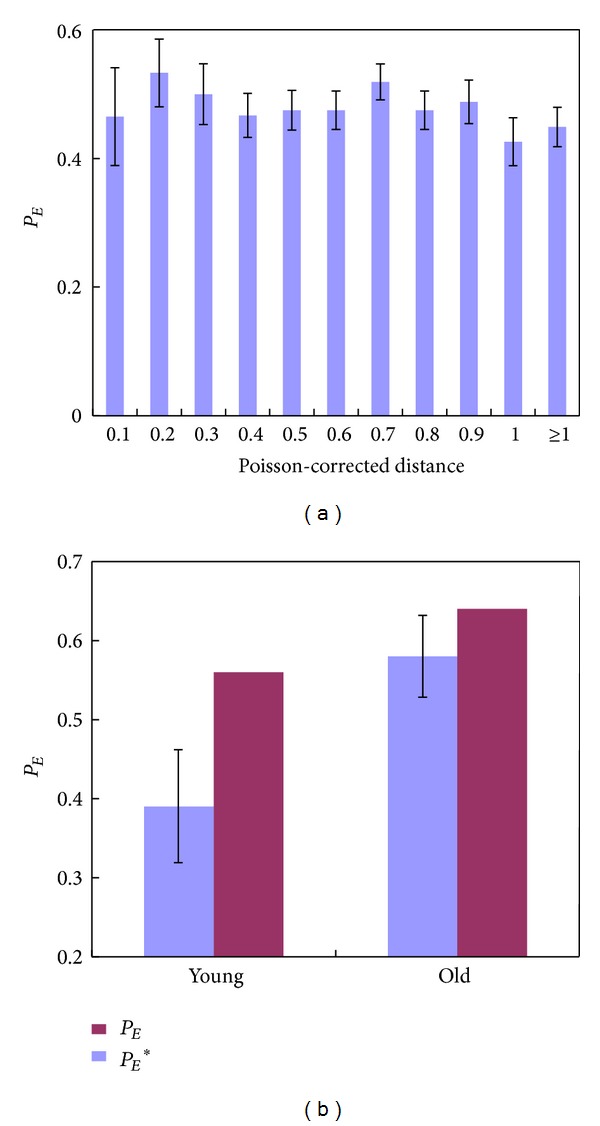
Relationship between *P*_*E*_ and protein sequence divergence. (a) *P*_*E*_ in duplicate genes is not correlated with the Poisson-corrected distance (*d*) between the target gene and its closest paralog in the genome. Error bars show one standard error. (b) *P*_*E*_ and *P*_E_* of mouse knockout duplicate pairs with sequence divergence *d* < 0.2. “Young” group represents the knockout genes with duplication age <310 mya, and “old” group represents the knockout genes with duplication age ≥310 mya. *P*_E_* is calculated based on the mean *d*_*N*_/*d*_*S*_ ratio for each group and the linear regression equation of Figure 3(a).

The protein sequence divergence between duplicate genes, or the evolutionary distance (*d*), was widely used as a proxy measure of the age of duplicates. In our study we used the Poisson-corrected method to estimate the protein sequence distance (*d*).  Figure 6(a) shows no correlation between*P*_*E*_ and* d*, as claimed in [10]. A straightforward explanation is that the sequence distance between duplicates (*d*) is determined by *d* = 2*vt*, where *v* is the evolutionary rate of the protein sequence and* t* is the age of duplicates. As shown in Figure 3(b), an ancient duplicate gene (a large* t*) tends to be conserved (low* v* as measured by low *d*_*N*_/*d*_*S*_ ratio) so that the *P*_*E*_-*d* independence could be the result of canceled *P*_*E*_-*t* and*P*_*E*_ *-d*_*N*_/*d*_*S*_ correlations.

Our conclusion that the *P*_*E*_-*d* relationship is not fundamental differs from Liao and Zhang [10]. Assuming that it is the protein sequence similarity, not the age of gene duplication, which determines the likelihood of compensation between duplicates, the authors of [10] argued that the lack of correlation between *P*_*E*_ and* d* may indicate the negligible role of duplicate genes in the mouse genetic robustness. Here, we conduct a simple case-study to show that it may not be the case. We divided 135 mouse KO duplicate pairs with *d* < 0.2 (corresponding to 82% sequence identity between KO duplicates and their paralogs) into the “young” group (age <310 mya, after the bird-mammal split) or the “old” group (≥310 mya). Strikingly, we found *P*_*E*_ = 0.39 for the young group and *P*_*E*_ = 0.58 for the old group (*χ* ^2^ = 4.56,* P* = 0.03) (Figure 6(b)). Moreover, we calculated the mean sequence conservation (the *d*_*N*_/*d*_*S*_ ratio) in both groups: *d*_*N*_/*d*_*S*_ = 0.12 for young duplicates and 0.02 for ancient duplicates. Does this mean that different *P*_*E*_ in young and old groups is caused by the difference in sequence conservation? From the*P*_*E*_ *-d*_*N*_/*d*_*S*_ regression in singletons (Figure 3(a)), we predict that, if there is no functional compensation between duplicates, the young group should have the *P*_*E*_ = 0.56 versus the old group *P*_*E*_ = 0.64 (Figure 6(b)), which is contradictory to our observation. We therefore conclude that, for these duplicate pairs with >82% protein sequence identity, recent duplicate pairs are functionally more compensated than ancient pairs.

The A&B model we proposed suggests that the age of gene duplication plays an important role in functional compensation between duplicates, while the sequence conservation indicates the likelihood of a duplicate gene actually genetically buffered by other (nonhomologous) genes, as supported by recent double deletions of yeast duplicate pairs [29, 30]. Noticing that, in many cases, the sequence similarity and functional similarity between paralogs may not be strongly correlated [31], we tentatively propose the transient hypothesis for the observed *P*_*E*_-age correlation. That is, because only a few nucleotide substitutions are responsible for the compensation loss between duplicates, the time interval for maintaining the effective compensation between duplicates mainly depends on the “waiting time” for these substitutions to occur.

## 3. Discussion 

In this study, we formulated an evolutionary model (A&B model) to address the knockout duplicate puzzle in mouse. That is, a duplicate gene to be essential depends on two mechanisms: the effect of genetic buffering (*g*) and the age-dependent effect of duplication compensation. We convincingly showed that the role of duplicates in mouse genetic robustness is nontrivial, similar to other simple model organisms [1–4]. There are substantial segmental or tandem gene duplications in the mouse genome around the mammalian radiation or even during the rodent lineage. These recently duplicated genes are expected to play major roles in the mouse gene robustness [11]. In spite of the fact that they were considerably underrepresented in the current mouse KO database, after the careful analysis that ruled out the potential confounding effect from sequence conservation, protein connectivity, functional bias, or bias of WGD duplicates, we reached the conclusion that differs sharply from the previous statement [10] of negligent duplicate effect on mouse genetic robustness. It is interesting to find that *P*_*E*_ seems to increase with organismal complexity. That is, though a greater fraction of genes in complex organisms may have been essential to ensure viability and fertility than that in simple organisms, for example, under laboratory conditions, *P*_*E*_ is ~7% in* Escherichia coli* [32], 17% in yeast [8, 33], and >46% in mouse, the age-dependent effect of duplicates on gene robustness remains similar from simple to complicated organisms. Of course, a more complete mouse KO database is crucial for further investigation.

Although there is no big difference between mouse and yeast in the role of duplicate genes in genetic robustness, mouse genetic robustness indeed reveals some unique features deserving further investigations: (i) why the *P*_*E*_ of mouse WGD duplicates is larger than the *P*_*E*_ of average single-copy mouse genes, but, in yeast, it is much smaller than its counterpart; (ii) why the *P*_*E*_ of yeast singletons is much larger than the *P*_*E*_ of duplicates, but the difference is not very evident in mouse even after controlling the sampling bias; (iii) why protein connectivity is high in mouse duplicated genes, in contrast to the case in the yeast [9, 14]. Though one may speculate that each problem may have several possible explanations, we propose a unified evolutionary model that can interpret these observations, which is the quite different age distribution of duplicated genes between mouse and budding yeast resulting from different evolutionary origins.

In the yeast* Saccharomyces cerevisiae*, the most recent WGD event occurred relatively recently (in the last ~100 million years) [34]. The majority of the yeast duplicated genes are quite young. For example, we found that only 13.1% of the yeast duplicates were generated 500 mya. In contrast, 58.9% of the mouse duplicates were created 500 mya (unpublished data). As shown in Figure 1, a significant portion of duplicate genes in vertebrates, including fishes, birds, and mammals, were generated by large-scale genome-wide duplications in the early stage of vertebrates [26, 35–39]. Though there still remains some controversies on how many rounds of WGDs had occurred during the evolution of early vertebrates, a general agreement has been reached that these duplication events may result in concomitant increase of developmental genes involving signal-transduction and transcription regulation that may be relevant to the expansion of cell types in the origin of vertebrates. For instance, we found a significant increase of paralogous genes in GPCRs (G-protein coupled receptors) and GPCR-pathway related protein families during the early stage of vertebrates. Transcription factors and protein kinases also show the same pattern [40]. These signaling-related molecules apparently tend to have more numbers of protein-protein interactions; many of them actually act as hubs in the process of signaling. If the evolutionary process of transition from invertebrate to vertebrate required the increase of tissue-specific signaling pathways, signaling-related duplicate genes may be favorably preserved in the genome. This hypothesis explains why protein connectivity in mammals is high in duplicate genes.

Another intriguing observation is the specific features of ancient duplicates. We found that ancient duplicates tend to be more conserved, and the ancient duplicate gene tends to be more essential than an average single-copy gene. First thought for why ancient duplicates are more conserved is puzzling, because it is generally believed that duplicated genes may have experienced a relaxed evolution due to the functional redundancy. Hence, an interpretation based on positive selection could be that the follow-up neofunctionalization may impose stronger functional constraints on these ancient duplication genes. Though it stands as an interesting hypothesis, we offer a much simpler explanation. For those ancient duplicate genes originated over 500 mya, only highly conserved duplicate pairs can be detected by the standard homologous search. In other words, sequence similarity between ancient duplicate genes with relatively low sequence conservation may be too low to be detected. Our simple calculation has shown that it may occur very likely. Suppose that the evolutionary rate of a gene is typically 3 × 10^−9^ per change/year. Since the ancient duplication event (500 mya), the sequence identity between duplicate copies, under the simplest Poisson model, is estimated to be exp⁡[−2 × 3 × 10^−9^ × 500 × 10^6^] = *e* ^−3^ ≈ 0.0498! Note that the cutoff for sequence similarity in homologous search is usually around 0.25. An interesting explanation for why ancient mouse duplicates even have a higher degree of gene essentiality than the average of singletons invokes acquisition of new functions that facilitates the loss process of functional compensation between duplicates. However, our analysis (Figure 3(c)) shows that a nonadaptive alternative may be more likely; that is, ancestral genes for those duplicated in early or prior to vertebrates may have stronger sequence conservation. In this case, using the overall proportion of essential genes in singletons as a reference may be misleading.

Since functional compensation of duplicated genes has been found to play an important role in genetic robustness in various species, from simple eukaryote yeast to complicated mammal mouse, it is highly expected that the similar scenario holds in human. However, owing to the impossibility of getting the large-scale human gene KO phenotypic data, it is not possible to systematically verify this expectation. Recently, several studies showed evidences that disrupt duplicate genes have less phenotype effect in human genome, indicating a possible contribution of duplicate genes to the human genetic robustness. For example, two separate studies found that the human specific nonprocessed pseudogenes or long-established lost genes are overrepresented in genes belonging to large gene families, such as olfactory receptor or zinc finger protein family [41, 42]. These results might indicate that loss of duplicate genes could be compensated by their close paralogous genes. Similarly, through a large-scale experimental survey of nonsense SNPs in the human genome, Yngvadottir et al. (2009) discovered 99 genes with homozygous nonsense SNPs in healthy human population. These genes could be considered as nonessential genes [43]. They found that 51% of nonessential genes have at least one paralog, whereas in comparison only 35% of all human genes are reported to have a paralog (*P* < 0.05). So, it is possible that their function is “backed up” by duplicated paralogs in the human genome. Moreover, Hsiao and Vitkup (2008) found that genes with close homologs are significantly less likely to harbor known disease mutations compared to genes with remote homologs [8]. In addition, close duplicates affect the phenotypic consequences of deleterious mutations by making a decrease in life expectancy less likely. If all the gene samples of above studies represent the entire genome, the results would mean that the effect of duplicate genes on genetic robustness holds in human genome.

In our study, the duplication age was estimated between the mouse KO gene and its closest paralog. Many mouse KO genes have more than one paralog, consisting of a large gene family. In such cases the pattern of functional compensation is complex, which cannot be revealed because most members have no KO phenotype information. Our approach is based on the premise that the closest paralog is the major determinant of functional compensation. Of course our treatment could be biased, and the future study should be gene-family based. The bottleneck still is the lack of sufficient KO genes. We indeed conducted a preliminary survey of the distribution of KO genes in a family but the dataset is too small to be useful at the current stage. Another technical issue is about the age of singleton. While we use the common procedure to determine singletons, the age of gene does affect *P*_*E*_ in both duplicate and singleton genes. One may see Chen et al. (2012) for details [44].

The mouse KO database provides a valuable resource to study the genomic features of vertebrate evolution from gene essentiality [9, 10, 45] to pleiotropy [46]. Since mouse tissue-specific developmental genes were largely duplicated in the early stage of vertebrates (~500 mya), while mammalian character-related genes were duplicated recently, the contribution of duplicates to genetic robustness may be more associated to mammalian-specific phenotypes. On the other hand, duplication events in the early stage of vertebrates were tightly associated with the expansion of signaling pathways for the evolution of vertebrate-specific multicellularity [16]. This may explain why gene duplicability and protein interactions are positively correlated [9], as signaling-related proteins tend to have high number of protein interactions. The effect of gene duplications on genetic robustness depends on the distribution of young duplicate genes in the current genome. Therefore, its impact varies among species, mainly because each species has its unique age distribution of gene duplications. For instance, due to recent polyploidizations, duplicate genes may dominate the genetic robustness in plant genomes [47]. It will be interesting to see whether the conclusions made in mouse hold in general when more invertebrate null mutation phenotypic data become available for such analyses.

## 4. Materials and Methods

### 4.1. Genomic Data

Protein sequences of mouse (NCBIM36), human (NCBI36), chicken (WASHUC2), and zebrafish (Zfish6) genes were extracted from Ensembl (release 59). If a gene had more than one alternative-splicing form, the longest isoform was used. Since several processed pseudogenes inserted into the genome very recently could be erroneously annotated as functional genes in Ensembl [48], we identified the single-exon genes with protein sequence identity ≥98% to multiple-exon genes as processed pseudogenes. The identified processed pseudogenes were excluded in the following analysis. The transcript and exon data of mouse genes were also obtained from Ensembl. For each alternatively spliced gene, the exon number was defined as the largest exon number of its all transcript isoforms.

Mouse phenotype and genotype association file (MGI_PhenoGenoMP.rpt) was downloaded from Mouse Genome Informatics (ftp://ftp.informatics.jax.org) (release 08/23/2010) [49]. This file contains specific mammalian phenotype (MP) ontology terms annotated to genotypes. Mammalian phenotype browser (http://www.informatics.jax.org/searches/MP_form.shtml) was used to match MP terms and phenotype details. Here, an essential gene was defined as a gene whose KO phenotype is annotated as lethality (including embryonic, prenatal, and postnatal lethality) or infertility [9]. We excluded all the phenotypic annotations due to multiple gene KO experiments and only used those of null mutation homozygotes by target deletion or gene-trap technologies. Totally, 4123 genes with phenotypic information were extracted from this file. We then classified these genes into 1921 essential genes and 2202 nonessential genes. Some different criteria were used to examine the effect of the definition of  “essential genes” that we used above. For example, we followed the methods of [10, 45] to define essential genes. We found that though *P*_*E*_ varies under different criteria for essential genes, it does not change our major results qualitatively (data not shown).

Homology information of mouse-human genes (mouse-rat) was obtained from Ensembl BioMart (release 59). The number of synonymous substitutions per synonymous site (*d*_*S*_) and the number of nonsynonymous substitutions per nonsynonymous site (*d*_*N*_) between mouse and human orthologs were estimated by the maximum likelihood method using PAML [50] and were retrieved from Ensembl EnsMart. For mouse genes have many human (rat) orthologs, the pair with the smallest *d*_*N*_/*d*_*S*_ ratio was used for further analysis.

We calculated the protein connectivity (*k*) based on the protein-protein interaction data of one-to-one human orthologous genes (including both yeast two-hybrid and literature-curated interactions, but excluding self-binding interactions) [26]. Because of the absence of the large-scale mouse protein-protein interaction experiment and the function similarity between human-mouse orthologous genes, here we use the protein connectivity of corresponding human orthologs to approximately represent that of the mouse KO genes.

### 4.2. Identification of Duplicate Genes and Singletons

We used a method similar to that of Gu et al. [51] to identify duplicate genes and single-copy genes. Because we want to detect the differences in *P*_*E*_ between real duplicates and singletons, we use stringent criteria to define duplicate genes and singletons. Briefly, every protein was used as the query to search against all other proteins by using Blastp (*E* = 1*e* − 10) [52]. Two proteins are scored as forming a link if (1) the alignable region between them is >80% of the longer protein and (2) the identity (*I*) between them is* I* ≥ 30% if the alignable region is longer than 150aa and *I* ≥ 0.01*n* + 4.8*L* ^−0.32[1+exp⁡(−*L*/1000)]^ for all other protein pairs, in which* n* = 6 and* L* is the alignable length between the two proteins. We deleted proteins if they formed a hit due to the presence of a repetitive element of the same family. The Blastp non-self best hit of a duplicate gene was defined as its closest paralog. A singleton gene is defined as a protein that does not hit any other proteins in the Blastp search with *E* = 1*e* − 10; this loose similarity search criterion was used to make sure that a singleton is indeed a singleton. Our results were essentially unchanged when we chose an even looser criterion, such as *E* = 1*e* − 5.

### 4.3. Dating Duplication Time of Mouse Duplicate Genes

**Figure 7:**
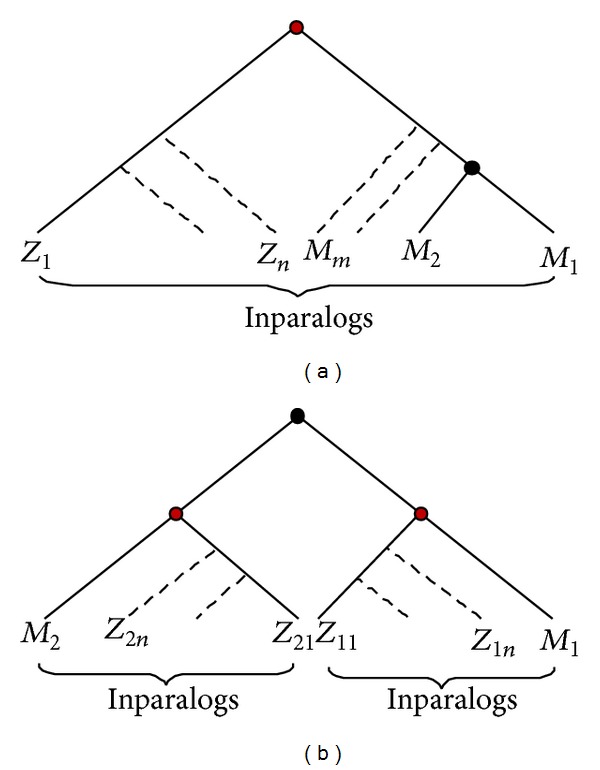
Illustration of the evolutionary relationship between mouse and zebrafish genes. Mouse duplicate events may occur after mouse-zebrafish split (a) or before it (b). Red node represents the speciation event and black node represents the duplication events. Genes under a red node represent a mouse-zebrafish inparalog cluster.

We developed an analytical pipeline to estimate the duplication times (ages) of mouse duplicate genes on a large scale, using the split-time between the mouse and zebrafish (430 million years ago, mya) as a calibration. First, we shall define* Inparalogs clusters of mouse and zebrafish*; that is, those paralogs duplicated after the mouse-zebrafish split, in either mouse or zebrafish lineage. One may see Figure 7 for illustration. Apparently, there are two modes for each duplicate pair: duplicated after the mouse-zebrafish split (Figure 7(a)) or before mouse-zebrafish split (Figure 7(b)).

We used the Inparanoid program (Version 2.0) to infer Inparalogs clusters of mouse and zebrafish [53]. Mouse and zebrafish genes in the same cluster are then identified as orthologs. A multiple alignment including the mouse duplicate genes, their closest paralogs, and their Inparalogs clusters (orthologs) was obtained by Tcoffee [54]. For those clusters containing more than 10 mouse or zebrafish Inparalogs, to reduce the complexity of calculation, besides mouse duplicate pair, 10 mouse or zebrafish Inparalogs were randomly selected for further alignment. Poisson-corrected distances between duplicates (*d*_*m*_) or orthologs were calculated after all alignment gaps were eliminated.

In each case (a) or (b) (Figure 7), we calculated the distance between the mouse knockout duplicate and its closest paralog and the averaged distance between mouse and zebrafish orthologs, which can be easily converted to the geological time (million years ago) under the assumption of molecular clock [16]. By this method, the duplicate time between each of 9503 mouse genes and its closest paralog was estimated (whole genome set). Among them, 2260 genes were KO target genes (knockout set).

## Supplementary Material

Figure S1: The effect of sequence conservation on the relationship between *P_E_* and duplicattion age. The *d_N_*/*d_S_* ration of mouse-rat one-to-one orthologous gene pairs was used as gene evolutionary conservation measurement. (a) Relationship between *P_E_* in singletons and the evolutionary conservation of the gene, measured by the ratio of the nonsynonymous (*d_N_*) to synonymous (*d_S_*) nucleotide distances between the target gene and its rat ortholog. (b) Mean *d_N_*/*d_S_*dS ratio for each age bin of duplicates. (c) *P_E_* in each age bin of duplicates—*P_E_* (dup, t) and that of singletons with the same *d_N_*/*d_S_* ratio—*P_E_* ∗(t). (d) Ratio of *P_E_* (dup, t) and *P_E_* ∗(t) in each age bin of duplicates.

## Abbreviations

*P*_*E*_: Proportion of essential genes
Mya: Million years ago
GPCRs: G-protein coupled receptors
WGD: Whole genome duplication
SSD: Small-scale duplication.
