# Supplementary material for: Effect of Duplicate Genes on Mouse Genetic Robustness: An Update
Source: Biomed Res Int. 2014 Jul 10;2014:758672. doi: 10.1155/2014/758672 (PMC4119742; doi:10.1155/2014/758672)
Supplement: Supplementary file 1 — Figure S1: The effect of sequence conservation on the relationship between PE and duplicattion age. The dN/dS ration of mouse-rat one-to-one orthologous gene pairs was used as gene evolutionary conservation measurement. (a) Relationship between PE in singletons and the evolutionary conservation of the gene, measured by the ratio of the nonsynonymous (dN) to synonymous (dS) nucleotide distances between the target gene and its rat ortholog. (b) Mean dN/dSdS ratio for each age bin of duplicates. (c) PE in each age bin of duplicates—PE (dup, t) and that of singletons with the same dN/dS ratio—PE ∗(t). (d) Ratio of PE (dup, t) and PE ∗(t) in each age bin of duplicates. [file 758672.f1.pdf]

Supporting Information

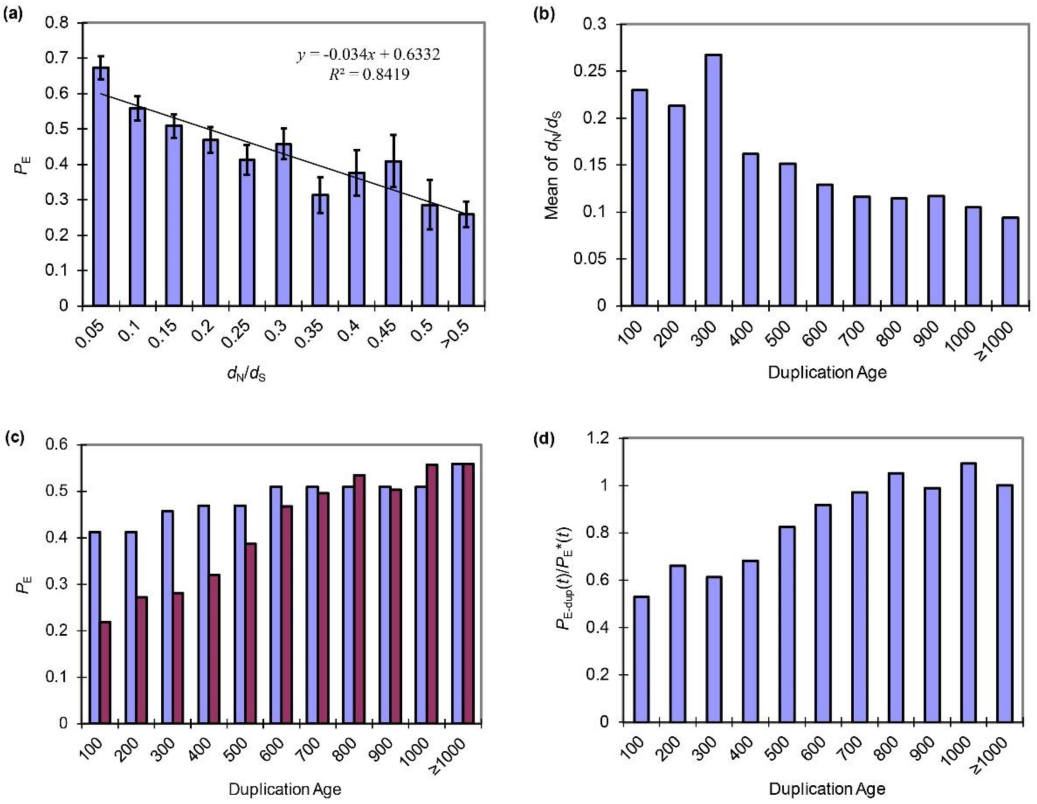

Figure S1. The effect of sequence conservation on the relationship between P_E and duplicattion age. The d_N/d_S ration of mouse-rat one-to-one orthologous gene pairs was used as gene evolutionary conservation measurement. (a) Relationship between P_E in singletons and the evolutionary conservation of the gene, measured by the ratio of the nonsynonymous (d_N) to synonymous (d_S) nucleotide distances between the target gene and its rat ortholog. (b) Mean d_N/d_S ratio for each age bin of duplicates. (c) P_E in each age bin of duplicates–P_E (dup, t) and that of singletons with the same d_N/d_S ratio–P_E *(t). (d) Ratio of P_E (dup, t) and P_E *(t) in each age bin of duplicates.
